# A new species of *Hemiptarsenus* Westwood (Hymenoptera, Eulophidae) from China, with a key to Chinese species

**DOI:** 10.3897/zookeys.1033.62129

**Published:** 2021-04-22

**Authors:** Shu-xia Tao, Kun Huang, Jing Tian, Chang-chun Ruan

**Affiliations:** 1 College of Plant Protection, Jilin Agricultural University, Xincheng Str. 2888, 130118, Changchun China Jilin Agricultural University Changchun China

**Keywords:** Agromyzidae, Chalcidoidea, *Chromatomyia horticola*, Eulophinae, parasitoids, taxonomy

## Abstract

A new species, *Hemiptarsenus jilinus* Tao, **sp. nov.**, is described and illustrated. All the type specimens were reared from *Chromatomyia horticola* (Goureau) (Diptera: Agromyzidae), a leafminer attacking the plants *Ixeris polycephala* Cass. and *Pterocypsela indica* (L.) Shih, in Jilin Province, north-eastern China. A key to Chinese species of the genus is provided.

## Introduction

*Hemiptarsenus* Westwood, 1833 (Hymenoptera: Eulophidae) contains 33 valid species worldwide (Noyes 2020), including seven species known from China (Sheng et al. 1989; Lee 1990; Zhu et al. 2000; Xu et al. 2001; Zhu and Huang 2002; Yang et al. 2015).

Leaf miners are serious pests of crops and ornamental plants worldwide (Spencer 1973). Parasitoids play an important role in inhibiting the occurrence of leaf miners (Gratton and Welter 2001). *Hemiptarsenus* includes numerous species which are potentially important for biological control of leaf miners belonging to Diptera, Lepidoptera, Coleoptera and Hymenoptera (Gibson 1997; Burgio et al. 2007; Yang et al. 2015).

Significant contributions to the taxonomy of this genus have been made by several authors, such as Bouček’s (1959) and Zhu and Huang’s (2003) studies for the Central European countries, Shafee and Rizvi’s (1988) and Narendran’s (2011) studies for the Indian fauna, Zhu et al.’s (2000) study for the Chinese fauna. In systematic studies at the generic level, Girault (1924) synonymised *Neodimmockia* Dodd, 1917 and *Hemiptarsenoideus* Girault, 1916; Schauff and LaSalle (1993) synonymised *Notanisomorpha* Ashmead, 1904; Bouček (1988) synonymised *Eriglyptoideus* Girault, 1913; Burks (2012) synonymised *Cleolophus* Mercet, 1924 and *Parpholema* Szelenyi, 1981 with the genus *Hemiptarsenus*.

In the present paper, a new species, which was reared from *Chromatomyia horticola* (Goureau) (Diptera: Agromyzidae), is described and a key to the known Chinese species of *Hemiptarsenus* is given.

## Materials and methods

All the specimens were reared from *Chromatomyia horticola* on rolled leaves of *Ixeris polycephala* Cass. (Campanulales: Compositae) and *Pterocypsela indica* (L.) Shih (Asterales: Asteraceae) from Jingyuetan National Forest Park of Changchun City, Jilin Province of China. Different host plants were placed in different insect cages, and each cage was labeled with the collecting date, locality, and host plant. The plants were maintained at 24–26 °C until emergence.

Photographs of the wings were taken with an OLYMPUS SZX16 stereomicroscope. Other photographs were taken with a KEYENCE VHX–2000 digital microscope. The type material of the new species was deposited in the Insect Museum of Jilin Agricultural University (**IMJAU**), Changchun, China.

The morphological terminology follows Yoder et al. (2010), Gibson (1997) and Bouček (1988) and the following abbreviations are used: F1–4, flagellar segments 1–4; SMV, submarginal vein; MV, marginal vein; PMV, postmarginal vein; STV, stigmal vein; POL, minimum distance between posterior ocelli; OOL, minimum distance between a posterior ocellus and corresponding eye margin. Absolute measurements in millimeters (mm) were used for the body and fore wing lengths. For all other dimensions, relative measurements were used.

## Taxonomy

### Hemiptarsenus

Taxon classificationAnimaliaHymenopteraEulophidae

Westwood, 1833

52CBDDF7-D91A-544E-ABEE-90EB1819C993

Hemiptarsenus Westwood, 1833: 122–123. Type-species: Hemiptarsenus fulvicollis Westwood

#### Diagnosis.

Torulus high on head, above lower margin of eye, hence apex of scape extending above level of vertex; funicle 4-segmented in female, and with 3 branches in male; notauli incomplete; axillae not angulately advanced; scutellum without sublateral grooves; median carina and plicae on propodeum nearly always indistinct or absent in majority of species; petiole distinct though not very long; fore wing and costal cell long and narrow, the fore wing at least 2.6 times as long as wide and costal cell 10–15 times as long as wide.

### Key to species of *Hemiptarsenus* Westwood from China (females)

**Table:** 

1	Propodeum elevated medially; plicae and median carina at least partly distinct	**2**
–	Propodeum sloping laterally; plicae or median carina absent	**5**
2	Propodeum less than half length of scutellum; mesosoma yellow with pronotum, mid lobe of mesoscutum, dorsellum, and median area between plicae and median carina dark	***H. strigiscuta* Zhu, LaSalle & Huang**
–	Propodeum about as long as scutellum; mesosoma completely green	**3**
3	Scutellum longitudinally sculptured; legs yellow with coxae and trochanters white	***H. jilinus* Tao, sp. nov.**
–	Scutellum reticulate; legs completely yellow	**4**
4	Petiole at least as long as wide; metafemora dark	***H. unguicellus* (Zetterstedt)**
–	Petiole short, transverse; metafemora yellow	***H. tabulaeformisi* Yang**
5	PMV shorter than or at most as long as STV, fore wing with disc slightly clouded	***H. fulvicollis* Westwood**
–	PMV 2× length of STV, fore wing hyaline	**6**
6	Scutellum reticulate; mesoscutum with transverse, yellow patch	***H. zilahisebessi* Erdös**
–	Scutellum longitudinally sculptured; mesoscutum completely metallic green	**7**
7	Mesosoma with scutellum orange-yellow or yellow	***H. ornatus* (Nees)**
–	Mesosoma completely metallic green	***H. varicornis* (Girault)**

### Hemiptarsenus jilinus

Taxon classificationAnimaliaHymenopteraEulophidae

Tao sp. nov.

C3544689-8DFF-5A0A-88F4-1074E636FE6C

http://zoobank.org/4D489171-EEA6-4DA1-9F40-02F7E9F996F3

Figs 1 , 2 , 3–10

#### Material examined.

***Holotype*** ♀ (IMJAU), China: Jilin Province, Jingyuetan National Forest Park of Changchun City (43°79.32'N, 125°45.23'E), 3–9 July 2019, reared by Rui-Jie Wang from *Chromatomyia horticola* (Goureau) (Diptera: Agromyzidae) on rolled leaves of *Ixeris polycephala* Cass. and *Pterocypsela indica* (L.) Shih.

***Paratypes***: 2♀ and 1♂ (IMJAU), same data as holotype.

#### Diagnosis.

The new species is easily distinguished from the other known members of the genus by the following combination of characters: head and mesosoma dark metallic green; back of gaster brown with a large yellowish patch near base, ventral panel of gaster yellow, apex brown; antennae (Fig. 3) with funicle dark brown, scape and pedicel pale yellow, clava uniformly white and 2-segmented; legs yellow with coxae and trochanters white; scutellum longitudinally sculptured, longer than mesoscutum; dorsellum raised-reticulate; propodeum shorter than scutellum, with median carina and plicae complete (Fig. 7).

#### Description.

**Figure 1.**
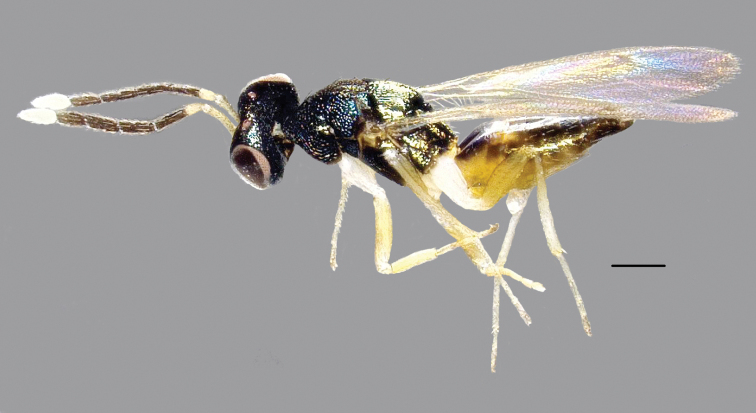
*Hemiptarsenus jilinus* sp. nov., female, holotype, lateral habitus. Scale bar: 200 µm.

**Female**, holotype (Fig. 1). Body length 1.68 mm, fore wing length 1.48 mm. Head and mesosoma dark metallic green. Ocelli and eyes red-brown. Antenna (Fig. 3) with funicle dark brown, scape and pedicel pale yellow. Funicle, scape and pedicel with brown setae; clava, including setae, white. Back of gaster brown with a dumbbell-shaped large yellowish patch near base, ventral panel of gaster yellow, apex brown. Legs yellowish with coxae and trochanters white. Wings hyaline with veins yellowish-brown. Callus with long, white setae.

***Head*** in dorsal view 2.5× as wide as long, micro-reticulate, with sparse short and brown setae. POL 1.6× OOL. Head in frontal view nearly quadrate (Fig. 5), 1.1× as wide as high. Eyes bare and oval, 1.4× as long as wide. Malar space 0.4× length of eye, malar sulcus straight and obvious. Lower margin of torulus located distinctly above lower margin of eye. Distance between toruli 0.3× diameter of torulus, 0.2× distance from torulus to eye margin. Antenna (Fig. 3) with scape slender and cylindrical, 8.2× as long as wide, extending far beyond vertex; pedicel 1.8× as long as wide and scape 6.3× as long as pedicel; funicle 4-segmented, F1 2.9× as long as pedicel. Ratio of lengths of F1–4 = 1.1:1.3:1.2:1.0, segments subequal in width. Funicle with numerous longitudinal sensilla. Clava 2-segmented, basal segment 1.6× as long as distal one.

***Mesosoma*** (Figs 6, 9) with coarse and raised reticulation dorsally and laterally, 1.6× as long as wide. Pronotum with 1 pair of black bristles. Mesoscutum (Fig. 6) slightly convex, mid lobe of mesoscutum with 2 pairs of black bristles. Notaulus inconspicuous. Scutellum longitudinally sculptured, longer than mesoscutum, with 2 pairs of stout, black bristles. Axilla micro-reticulate. Dorsellum narrow and reticulate. Propodeum (Fig. 7) shorter than scutellum, with median carina and plicae complete, propodeal spiracle small and round, callus densely setose. Middle area of propodeum between two plicae slightly elevated. Lateral and ventral panel of pronotum and prepectus with coarse reticulate sculpture. Fore wing (Fig. 8) 2.6× as long as wide. Costal cell 13.3× as long as wide, with a row of brown setae. Speculum present, but small. SMV with 6 setae on dorsal surface. Relative lengths of veins SMV:MV:PMV:STV = 15:19:9:5. Several admarginal setae present below MV. Speculum closed and basal setal line present. Precoxae with several long, white setae. Apices of pre- and mesofemora with a black spur. Femora, tibiae and tarsi of all legs with a few rows of short brown setae. Apices of tibiae of all legs with a tibial spur. Metacoxae with several short, black setae.

***Metasoma*** (Fig. 10). Elongate-ovate in dorsal view, 1.8× as long as wide and about as long as head plus mesosoma, apex of gaster acute. Petiole short, transverse, barely visible in dorsal view. Tergites smooth, with sparse short, pale setae. Ratio of lengths of tergites = 7.0:2.5:3.0:3.5:4.0:2.0. Cercal plate with two dark setae of subequal length. Third valvula slightly exerted at apex of gaster.

**Figure 2.**
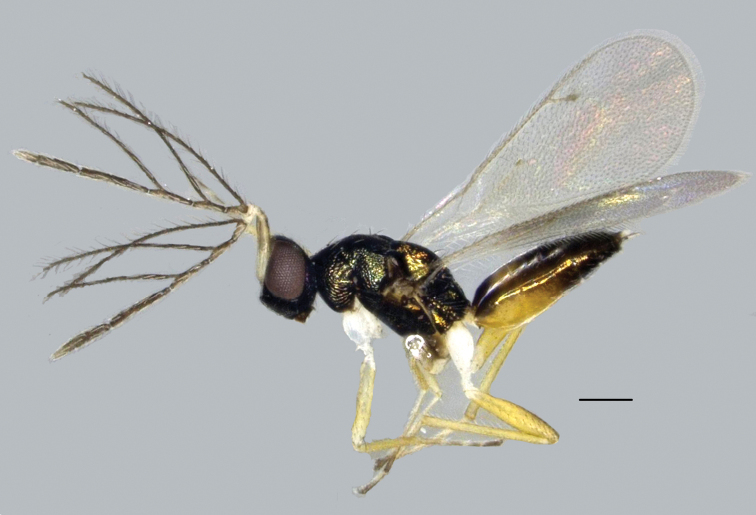
*Hemiptarsenus jilinus* sp. nov., male, paratype, lateral habitus. Scale bar: 200 µm.

**Male** (Fig. 2). Sexual dimorphism evident and smaller than female. Body length 1.61 mm, fore wing length 1.45 mm. Antennae (Fig. 4) with flagellum dark brown, funicle with 3 long branches, with long setae. F1 1.3× as long as pedicel. Ratio of lengths of F1–4 = 1.0:1.6:2.2:3.6. Last tarsomeres brown. Back of metasoma with a semicircular yellowish patch near base. Apex of metasoma obtuse. Genitalia protruding in dorsal view.

#### Variation.

Apart from the different body sizes of specimens, the main variation is in the color. Back of scape and pedicel pale brown to yellowish; scutellum green with green metallic tinge to blue-green with purple metallic tinge; back of hind femora pale brown to yellowish.

#### Biology.

The new species was reared from *Chromatomyia horticola* on rolled leaves of *Ixeris polycephala* and *Pterocypsela indica* Shih in Jingyuetan National Forest Park, Changchun City, where the vegetation is coniferous and broad-leaved mixed forest. The sampling site is slightly disturbed by occasional tourism.

#### Distribution.

China (Jilin).

#### Etymology.

The specific name is derived from the type locality’s province name, Jilin Province.

#### Remarks.

The new species is similar to *H. aditus* Narendran, 2011 in the general appearance, but differs from the latter in having: 1) Pedicel of antennae pale yellow (black in *H. aditus*); 2) Clava 2-segmented (1-segmented in *H. aditus*); 3) Dorsellum raised-reticulate (mostly smooth and shiny in *H. aditus*). 4) Propodeum with complete median carina (median carina absent in *H. aditus*) (Narendran 2011).

**Figures 3–10.**
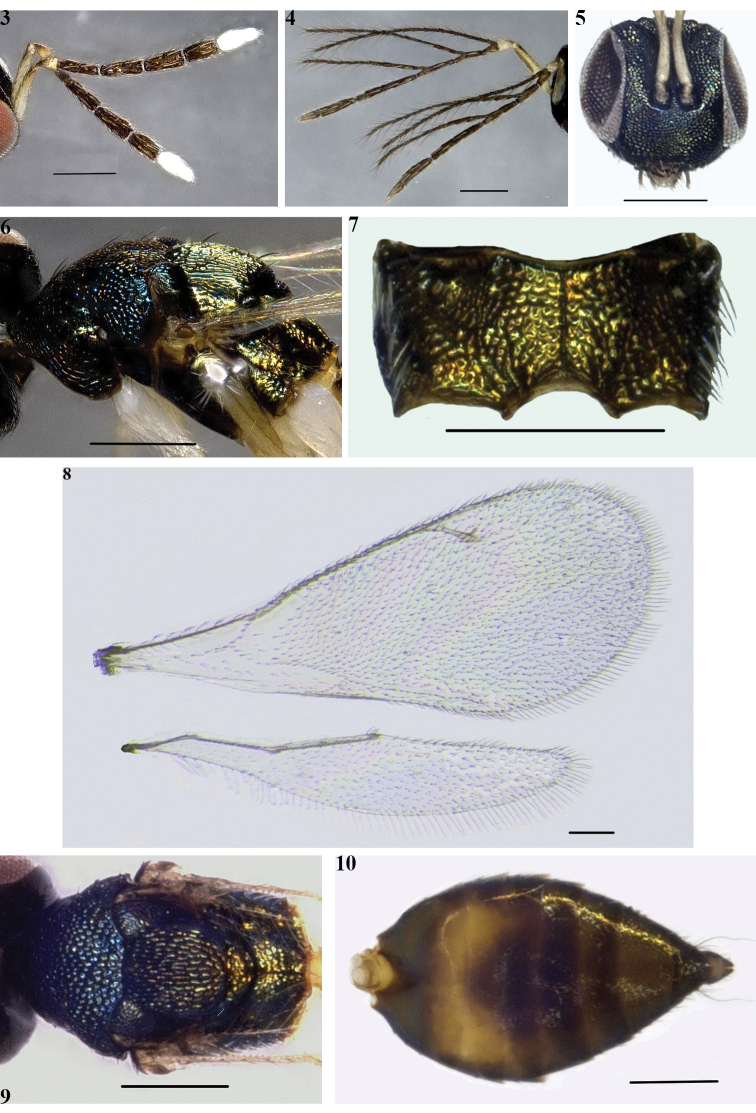
*Hemiptarsenus jilinus* sp. nov., female (**3, 5–10**) male (**4**) **3** antenna **4** antenna **5** head, anterior view **6** mesosoma, lateral view **7** propodeum, dorsal view **8** wings **9** mesosoma, dorsal view **10** metasoma, dorsal view. Scale bars: 200 µm (**3–10**).

## Discussion

In China, there are seven known members of *Hemiptarsenus*, with hosts and distributions as follows: *H. varicornis* Girault, 1913, *H. unguicellus* Zetterstedt, 1838, *H. ornatus* Nees, 1834, *H. zilahisebessi* Erdös, 1951 and *H. fulvicollis* Westwood, 1833 parasitize various species and are widely distributed (Sheng 1989; Wen et al. 2000; Zhu et al. 2000; Xu et al. 2001; Yao 2005; Pan 2019); *H. tabulaeformisi* Yang *in*Yang et al. 2015 parasitizes *Dendrolimus tabulaeformis* Tsai & Liu (Lepidoptera: Lasiocampidae) and is distributed in Beijing City (Yang et al. 2015); *H. strigiscuta*Zhu et al. 2000 is distributed in Hunan and its hosts are unknown (Zhu et al. 2000).

## Supplementary Material

XML Treatment for Hemiptarsenus

XML Treatment for Hemiptarsenus jilinus
